# Facilitation of the mental health of adolescents abusing substances: A concept analysis

**DOI:** 10.4102/hsag.v29i0.2343

**Published:** 2024-02-27

**Authors:** Tinyiko N. Rikhotso, Mary Maluleke, Ndidzulafhi S. Raliphaswa, Thingahangwi C. Masutha, Mphedziseni E. Rangwaneni

**Affiliations:** 1 Department of Advanced Nursing Sciences, Faculty of Health Sciences, University of Venda, Thohoyandou, South Africa; 2 Department of Nursing, Faculty of Health Sciences, University of Johannesburg, Johannesburg, South Africa; 3 Department of Advanced Nursing Sciences, Faculty of Health Sciences, University of Johannesburg, Johannesburg, South Africa

**Keywords:** concept analysis, facilitation, adolescents, substance abuse, mental health

## Abstract

**Background:**

The use and abuse of substances, especially among the youth, continues to be of serious concern within the international community. This behaviour affects them as individuals, their families, the community in which they live, and society at large. Findings from a study conducted by the researcher indicate that adolescents who abuse substances experience a range of emotional, physical, academic and social setbacks, and challenges. Ultimately, the mental health of these adolescents is affected.

**Aim:**

The study aims to identify and define the central concept ‘facilitation of mental health’ of adolescents abusing substances.

**Setting:**

The researcher’s minor dissertation, whereby the lived experiences of adolescents abusing substances were explored. The basic elements, structure and functions were examined and analysed.

**Methods:**

The concept’s basic elements, structure and functions were examined, followed by an analysis and reasoning strategies to define the central concept. The process was implemented over two phases.

**Results:**

The central concept ‘facilitation of mental health’ of adolescents abusing substances was identified and defined using dictionary and subject definitions.

**Conclusion:**

The identification and definition of the central concept is an important part of developing a model as a frame of reference for psychiatric nurses to facilitate the mental health of adolescents abusing substances.

**Contribution:**

The findings of the study would assist in the development of a model as a frame of reference for psychiatric nurses to facilitate the mental health of adolescents abusing substances.

## Introduction

The use and abuse of substances, especially among adolescents continues to be a serious concern within the international community. This behaviour affects adolescents as individuals, their families, the community in which they live, and society at large. Substance abuse causes social and family disorganisation, economic instability, social insecurity, and curbs progress for individuals, families, and the State. Some effort towards intervening in this challenge, focussing on mental health promotion and substance abuse prevention was made, yet it appears it was not enough (Steel et al. 2020; World Health Organization [WHO] 2020). There are some shortfalls in these interventions as there is no satisfying improvement related to this phenomenon; instead, the prevalence of substance abuse is becoming worse each day (Cardenas et al. 2021; Setlalentoa, Ryke & Strydom 2016) Findings from the researcher’s minor dissertation (Rikhotso 2008) indicate that adolescents who abuse substances experience a range of emotional, physical, academic, and social setbacks and challenges.

Certain interventions concerning adolescents’ substance abuse were cited in another study (Salam et al. 2016) and included promoting sexual and reproductive health, nutrition interventions, immunisation, mental health promotion, substance abuse prevention and the prevention of unintended injuries. Another study conducted in the North-West Province of South Africa (Setlalentoa et al. 2016) proposed some interventions to address alcohol abuse. These called for holistic, multi-level and multi-sectoral interventions for all role-players, such as government departments, non-governmental organisations, the community, and faith-based organisations (Setlalentoa et al. 2016). Moreover, (Gallop et al. 2020) recommendations to reduce adolescent substance abuse included (Rikhotso 2008) health promotion activities, programmes, and services; (WHO 2020) constructive media-based public education campaigns; (Steel et al. 2020) development and implementation of school-based substance abuse programmes; and (Cardenas et al. 2021) provision of recreational facilities.

Among the interventions that were developed so far, according to the latter studies, it is evident that few had minimal impact while most of them had no effect at all. The South African Nursing Council has not acknowledged the facilitation of mental health of adolescents abusing substances for inclusion in the scope of practice of psychiatric nurses. The researcher, therefore, conceived the idea that developing a model whereby psychiatric nurses who provide care for adolescents abusing substances while admitted in psychiatric institutions to facilitate their mental health would bridge the gap. Moreso, because the model would not be something that is imposed on the adolescents but a step-by-step interactive process between the psychiatric nurses and those adolescents.It would be a participatory interaction for both on the way towards achieving adolescent’s mental health. To develop such a model, the researcher had to identify the concepts from the previous study, (Rikhotso 2008) define and classify these concepts, and place them into relationship. As a result, this study of concept analysis to develop a model to facilitate the mental health of adolescents was conducted.

## Objective

The objective of this article is to define the central concept ‘facilitation of mental health’ of adolescents abusing substances.

## Research methods and design

This study utilised a qualitative, exploratory, descriptive, contextual approach and theory generating design, and was guided by a Post-modern Constructivist Philosophy of Science. This philosophy relies on two concepts: post-modernism and social constructivism. Post-modernism’s emphasis is on setting knowledge claims within the conditions of the world today and in the multiple perspectives of class, race, gender and other group affiliations (Creswell 2018). Social constructivism occurs through social and environmental interactions and emphasises that knowledge exchange between researchers and healthcare professionals must happen in a mutually created social context (Thomas et al. 2014).

According to Walker and Avant (2011), concept analysis is the process of examining the basic elements, structure, and functions of a concept. Rodgers, Jacelon and Knafl (2018) view concept analysis as a branch of empirical linguistics, as it contains the assumption that a definition of a term may be found that pertains to its representation in a natural language. The concept analysis process was undertaken during model development, since it provides precise theoretical and operational definitions for use in model development and research. It also enables the researcher to classify similar concepts.

The basic elements, structure, and functions of a concept were examined, following the theory development approaches, namely, analysis, synthesis, and inductive reasoning strategy (Martinez et al. 2021) to define the central concept. The identified central concept in this study was analysed to identify essential criteria to define it (Gomba 2017; Walker & Avant 2011). This study warranted the application of synthesis so that the broken down and sorted concepts from the researcher’s previous study (Rikhotso 2008) could be reconnected to form a meaningful and comprehensive whole (Leedy & Ormrod 2015; Walker & Avant 2011). The researcher followed the inductive reasoning approach whereby the conclusions that were developed through the phenomenological interviews and the observations, as reflected in the field notes, were drawn from the participants’ shared experience (Ravitch & Carl 2016; Tema 2017).

The process of concept analysis was implemented over two phases. Phase One involved identifying the central concept ‘facilitation of mental health’ of adolescents abusing substances, derived from the researcher’s minor dissertation (Rikhotso 2008), in which the lived experiences of adolescents were explored using semi-structured individual phenomenological interviews. Phase Two involved defining the concepts and placing them into relationship. Those took place through the use of theoretical and operational definitions obtained from dictionaries and subject sources.

### Ethical considerations

Ethical approval was received from the Faculty of Health Sciences Research Committee of University of Johannesburg on 15 November 2016 with the ethical clearance number REC-01-169-2016.

## Results

The results of Phase One helped the researcher identify the central concept, and in Phase Two, the central concept was defined and classified. The process unfolded as presented in the following sections:

### Phase one: Identification of the central concept

To identify the central concept, the researcher obtained the findings from her minor dissertation from which the central concept was derived. The minor dissertation focussed on substance abuse among adolescents in Limpopo Province (Rikhotso 2008). Five themes were identified. These are summarised in Table 1, including the respective direct quotes from the participants (Rikhotso 2008).

**TABLE 1:** Themes and direct quotes.

Theme	Quote
1. Adolescents’ substance abuse behaviour	‘I started drinking at an early age. I began when my father started to buy us liquor during special holidays like Christmas, Easter, New Year, and when there were ancestral celebrations at home.’ (Participant 1) ‘Even if I happen to be without money, as liquor is expensive, my friends with whom I drink share with me.’ (Participant 2) ‘He forces all of the family members to engage in Rastafarian activities such as smoking marijuana and keeping long hair which is styled into dreads.’ (Participant 7)
2. Adolescents’ motivation for continuing substance abuse	‘I need to tell my friends that I don’t want to drink again and say no, when they invite me to take rounds with them, but I still feel that they will laugh at me and I might lose them.’ (Participant 2) ‘I sometimes could crave for liquor when I am lonely and during weekends.’ (Participant 8)
3. Effects of substance abuse on the lives of adolescents	‘This whole thing makes me left in the middle without knowing what to do to come out of this problem.’ (Participant 8) ‘I also feel hopeless as it is now in many occasions that I tried to quit drinking but I am failing.’ (Participant 8) ‘I sometimes tell myself that it would be better if I was dead because my whole life is destroyed.’ (Participant 5) ‘My father does not trust me anymore.’ (Participant 6)
4. Factors affecting adolescents’ discontinuation of substance abuse	‘I see myself as a slave of beer. I want it with my whole heart to quit drinking.’ (Participant 1) ‘But since I got used to sniffing glue, I cannot control it. I frequently crave for it and make sure I get it.’ (Participant 3) ‘If I can leave smoking my mother can be very happy.’ (Participant 6)
5. Adolescents’ plan of action to discontinue substance abuse	‘I want to quit drinking and show her that I can still say the same words even when I’m not drunk.’ (Participant 4) ‘I will reduce the cigarettes I used to smoke per day bit by bit. I will also talk to my father to buy me nicotine tablets so that when I crave for tobacco, I chew them.’ (Participant 6) ‘I think that if I can be able to leave drinking, my life can be fine again.’ (Participant 8)

*Source*: Rikhotso, T.N., 2008, ‘Substance abuse among adolescents’, MCur Psychiatric Nursing minor-dissertation, University of Johannesburg.

Considering the findings from a study by conducted by Hlahla and Mothiba (2022), it is evident that adolescents need to be assisted for them to be mentally healthy. From the narratives, the researcher identified that adolescents engaged in substance abuse activities to self-medicate (Mianowski, Borodo & Schreiber 2019). Some adolescents were abusing substances to ease depressive feelings, others as a means of running away from the reality or fear of confronting their parents, avoiding rejection by peers, and having a feeling of belonging. There were also instances where research participants claimed they were abusing substances to find solutions to their problems; only to find that even if there were solutions, they were temporary, and their situation ultimately worsened.

The adolescents’ experiences of abusing substances influenced their decision-making abilities and choices. The fact that the interviews with adolescents took place in a psychiatric ward shows that their mental status was affected. Therefore, they had moved from mental health to the mental illness continuum, as seen by the disharmony between their thoughts, feelings, and actions (Perko & Kreigh 1988). Perko and Kreigh (1988) further point out that mental illness encompasses psychological, emotional, and social disequilibrium, as noted from the quotes. To strike a balance between these dimensions implies that the mental functioning of individuals needs to be facilitated in order for them to adjust and adapt to daily living. The participating adolescents further acknowledged their awareness of being in an unfavourable situation and seeking assistance. However, they did not know how to go about obtaining this assistance. As the statements were placed into relationship, the experiences by adolescents in relation to substance abuse confirmed that:

- Adolescents who abuse substances have causative reasons for starting to abuse substances.
- Adolescents have internal and external factors that make it difficult for them to stop abusing substances, even if they wish to.
- Adolescents’ lives are affected in different dimensions as they use different substances.
- There are some driving forces that initiate a zeal in adolescents to discontinue abusing substances.
- Adolescents themselves are not pleased with their substance abuse behaviour, thus making plans to discontinue.

From the verbatim, the essential and related concepts were derived (See Table 2). These were classified based on shared characteristics.

**TABLE 2:** List of central concepts, essential and related criteria.

Central concept	Essential criteria	Related criteria
Facilitation	To make easier	*Improvement* *Bridging of obstacles* *Help* *Intervene* *Enhance* *Support*
	Dynamic interactive process	*Conscious involvement* *Participation* *Create a positive environment* *Promotion of health* *Dialogue* *Sharing*
	Guide	*Empowerment* *To give advice* *Lead* *Manage* *Accompaniment*
Mental health	Maturity	*Identity development* *Reaching a point of equilibrium*
	Positive or spiritual emotions	*Feeling comfortable* *Excitement* *Interest* *Humour* *Sense of mastery*
	Emotional intelligence	*Self-awareness* *Empathy* *Establish and maintain meaningful relationships* *Goal-directed (self-motivation)* *Self-management* *Work productively and fruitfully*
	Subjective state of wellbeing	*Contentment* *Autonomy* *Responsibility for outcomes of decisions* *Emotional, psychological and social wellbeing* *Satisfaction and fulfilment in exercising and expanding own potential*
	Resilience	*Consciously seeking social support* *Conscious cognitive strategies* *Healthy adaptive involuntary coping mechanisms (humour, altruism, sublimation, suppression, anticipation)* *Ability to make adjustments.* *Confirm and follow a philosophy of life*

*Source*: Adapted from Rikhotso, TN., 2020, ‘A model for psychiatric nurses to facilitate the mental health of adolescents abusing substances’, *Doctoral thesis, University of Johannesburg*, viewed 15 November 2023, from https://ujcontent.uj.ac.za

The central concept was therefore identified as the ‘facilitation of mental health’ of adolescents abusing substances by psychiatric nurses as they are brought under their care.

### Phase two: The definition of the central concept

While defining the central concept, the researcher used sources that enabled the provision of a link between theoretical abstractions and empirical indicators (Chinn & Kramer 2017). For each of the related concepts, a dictionary definition, which provides synonyms and antonyms and conveys commonly accepted ways in which words are used (Chinn & Kramer 2017), was employed. A subject definition, which conveys meanings about the discipline’s domain from which the concept comes, was also incorporated (Chinn & Kramer 2017).

These concepts are ‘facilitation’ and ‘mental health’, respectively. As stated, dictionary (both hard copies and online) and subject sources (including psychiatric nursing, psychiatry and psychology) were used to obtain definitions for these concepts. During concept analysis, the essential criteria were written in Italics. Table 2 provides a summary of dictionary and subject definition of the concept ‘facilitation’ and ‘mental health’, in which the central, essential, and related criteria are indicated.

#### Dictionary definitions of the concept ‘facilitation’

Waite (2012) defines ‘facilitation’ as to make something easy or easier. The verb for ‘facilitation’ is ‘facilitate’, which is defined as to make an action or process possible or easier. Conversely, a ‘facilitator’ is defined as a person who helps somebody do something more easily by discussing problems, *giving advice*, and so forth, rather than telling them what to do. It is a thing that helps a process to take place (Hornby 2010). Free Dictionary (2021) presents ‘facilitation’ as the act of making easy or easier, or the state of being made easy or easier. According to Princeton WordNet Dictionary (2003–2012), ‘facilitation’ is the condition of being made easy; the act of making easier the progress, or *improvement* of something.

#### Subject definition of ‘facilitation’

‘Social facilitation’ is defined as the *improvement* of a group, produced by the mere presence of others (McAllister, Withyman & Knight 2018). Ntshingila et al. (2021) argue that the purpose of facilitation is to enable a group of people to achieve their own purpose in their own agreed way. They also describe group facilitation as an intuitive art form of moment-by-moment awareness that requires discipline. Facilitators frequently must act ‘in the moment’; that is, deciding if, when and how to *intervene* in group discussions (Ntshingila et al. 2021).

The facilitator is described as a gentle guide, making it easier for the group to reach its goals International Council on Archives (ICA) 2014. The facilitator must be fully present and transparent in the group (Ntshingila et al. 2021). The facilitator has the role of *empowering* the participants and ensuring that the facilitation of learning occurs through active and *conscious involvement* of the whole person (Downing 2012).

Facilitation, as defined by the Theory for Health Promotion in Nursing (University of Johannesburg 2017), is a dynamic interactive process for the *promotion of health* through the *creation of a positive environment* and the mobilisation of resources, as well as the identification and *bridging of obstacles*. Facilitation is concerned with *helping* people (individually or in groups) to work effectively and efficiently to achieve a particular goal or learning outcome. It is a combination of skills, techniques and art involving interaction with adolescents abusing substances to draw out their ideas and *lead* them to new meanings and understandings (Nkuna 2017).

Facilitation is increasingly being used as a *participatory* approach that *enhances* team effectiveness in *dialogue*, analysis, decision-making, planning, divergence, and convergence (Thorpe 2016). Thorpe (2016) further alluded that, for facilitation to yield the expected outcomes, the facilitator must have the following competencies:

- Set and maintain a shared group culture – the facilitator ensures that care and respect are present in interventions, uses behaviours and attitudes that enable greater participation by the group, shares valid information that enables participants to learn together, holds and *supports* the group in their culture, and works with others to set expectations for *participation* and *manage acknowledgements.*
- Plan and prepare – the facilitator develops appropriate *lead* times, prepares access to group technologies and their effective use, ensures that the right decision-making people are involved, and are timely and responsive to others.

The facilitation process can be described as a road on which the facilitator walks with the client, *accompanying* that client on the journey of self-discovery (Grobler, Schenck & Mbedzi 2013). A facilitated session is a highly structured meeting in which the meeting leader, who is the facilitator, **guides** the participants through a series of pre-defined steps to arrive at a result that is created, understood, and accepted by all the participants (Wilkinson 2012). The process makes it possible for participants to explore issues such as: ‘Who am I?’, ‘What are the thoughts, perceptions, feelings, needs, behaviours and values that make me who I am?’ and ‘What prevents me from being who I want to be or living to my full potential?’ The facilitator cannot walk this road for the participant. Only the participant knows what it feels like under his feet and how it feels to walk in his shoes, where he wants to go or where he is going, where he does not want to go, or which road is too steep for his legs.

Exforsys (2021) states that the facilitator guides participants to a learning journey to discover their own experiences and explore those of others, identify their strengths and weaknesses, and *share* what they already know with the rest of the group. In certain instances, the facilitator also *shares* knowledge apart from just guiding the group in the process. Effective facilitation skills must be demonstrated. This includes the ability to communicate, the ability to *manage* and *lead* a group, active listening skills, effective questioning skills, and the ability to easily resolve any conflicts and misunderstandings.

#### Dictionary definition of the concept ‘mental health’

Mental health includes *emotional, psychological*, and *social* wellbeing. It affects how an individual thinks, feels and acts in response to specific life situations. It also determines how an individual *handles stress, relates to others*, and *makes choices*. Mental health is important at every stage of life, from childhood, adolescence, through to adulthood (Medline Plus 2021). Mental health is a level of *psychological* wellbeing, or an absence of mental illness. It is the *psychological* state of someone *functioning at a satisfactory level* of *emotional* and *behavioural adjustment* (Smith 2021).

Mental health is the condition of being mentally and *emotionally* sound as characterised by the absence of mental illness and presence of adequate *adjustment,* especially as reflected in *feeling comfortable* about oneself, having positive feelings about others, and the *ability to meet the demands* of daily life (Merriam-Webster 2021). Free Dictionary (2021) defines ‘mental health’ as a state of *emotional and psychological* wellbeing in which an individual can use their *cognitive and emotional capabilities, function* in society, and *meet the ordinary demands* of everyday life.

Emotional intelligence is the measure of an individual’s *ability to recognise and manage their emotions*, and the *emotions of other people*, both individually and in groups. People with higher emotional intelligence find it easier *to form and maintain interpersonal relationships* and ‘fit in’ to group situations. They are also better at understanding their own *psychological* state, which can include *managing stress effectively*. A range of skills are elements of emotional intelligence, such as *self-awareness, self-management, self-motivation, empathy,* and *social skills* (Skills You Need 2021).

Beyond Blue (2021) is of the opinion that ‘mental health’ is often used as a substitute for mental health conditions, such as depression, anxiety disorders, schizophrenia, and others. It is about achieving and helping everyone reach their *full potential*. This will contribute to the prevention of mental health conditions and *support* people who have experienced these conditions to get as well as they can and lead a *full and contributing life*. Having *social connections, good personal relationships,* and being part of the community is vital to maintaining good mental health and contributing to people’s recovery, should they become unwell.

#### Subject definition of the concept ‘mental health’

World Health Organization (2014) defines ‘mental health’ as a state of wellbeing in which every individual *realises one’s own potential*, can *cope with the normal stresses* of life, can *work productively and fruitfully*, and is *able to make a contribution to own community*. It is further indicated that mental health, physical health, and social functioning are interdependent, and the relationship between these is complex. Mental health is defined as a state of subjective wellbeing whereby an individual’s *socio-emotional intelligence* and *positive* or *spiritual emotions* are above normal, as characterised by *resilience*, in which individuals *overcome stressful situations* by *developing defence mechanisms* to *cope* (Sadock, Sadock & Ruiz 2020).

Sadock et al. (2020) again added that there are six contrasting approaches to mental health. These include: (1) Mentally healthy people function above normal, meaning they are in a *reasonable state of functioning*. (2) The association of mental health and maturity is likely mediated by progressive brain myelination and the evolution of emotional and *social* intelligence through experience. In this model, adolescents must achieve an *identity* that allows them to become separate from their parents. The task of *identity* requires mastering the last task of childhood. (3) Mental and spiritual health is defined as the amalgam of the positive emotions that bind us to other human beings and involves *human connections*. These positive emotions appear to be common denominators of major faiths, some of which include *excitement, interest, contentment, humour,* and a *sense of mastery*. (4) Socio-emotional intelligence reflects above-average mental health. Anyone can become angry; that is easy. But socio-emotional intelligence is when one becomes angry with the right person, for the right reason, to the right degree, at the right time, and in the right way. (5) Mental health is subjective wellbeing. The maintenance of self-efficacy, agency, and *autonomy* makes additional environmental contributions to subjective wellbeing. This implies that not only does an individual impose joy on others, but also involves subjective happiness. Assuming *responsibility for favourable and unfavourable outcomes* is another main factor leading to subjective wellbeing. (6) Mental health as resilience involves three classes of defence mechanisms, namely *consciously seeking support* from others, *conscious cognitive strategies* that individuals use to *master stress,* and *healthy adaptive involuntary coping mechanisms* (humour, altruism, sublimation, suppression, anticipation) that distort perceptions of internal and external reality in order to reduce objective distress, anxiety and depression.

Mental wellbeing refers not only to physical health but also, and more importantly, to the mental and *social* state of an individual. Mental order and disorders are observed as cultural categories adopted through or embodied in *social interaction*. Wellbeing and disorder are themes of the utmost importance, since everyone is touched by various disorders or illnesses and healing, wellbeing, or lack thereof (Katajala-Peltomaa & Niiranen 2014).

Uys and Middleton (2014) define ‘mental health’ as an evolving process in which the individual’s internal demands and needs are brought into contact with the reality of the environment in which they live. Its achievement is obtained through *successful adaptation*, which requires an individual to *establish a point of equilibrium* between wants, needs, abilities, ambitions, values, and feelings. The real or perceived expectations of society and the surrounding environment influence the way in which the individual operates. Uys and Middleton (2014) talked about mentally healthy people being resilient, while Uys & Middleton (2014) said mentally healthy individuals use more *adaptive coping skills*.

Mental health status means the level of mental wellbeing of an individual as affected by physical, *social* and *psychological* factors, which may result in a psychiatric diagnosis (*Mental Health Care Act [MHCA] 17 of 2002*). Mental health recovery is a journey of healing and transformation, enabling persons with mental health problems to *live a meaningful life* in a community of their choice, while striving to achieve their *full potential* (Parker 2014).

Psychiatric mental health nursing is a specialised area of nursing practice committed to promoting mental health through the assessment of human responses to mental health problems and psychiatric disorders (Jones, Fitzpatrick & Rogers 2016). Mental health problems can be seen as a continuum varying from *normal reactions* to everyday events, to serious disability requiring long-term support. *Connecting with people*, being active, learning new things, acts of *altruism* and being *aware of oneself* is evidenced as ways of promoting our wellbeing, but mental order remains rather more loosely defined than mental disorder (Kinsella & Kinsella 2015).

Mentally healthy persons possess the ability to make *adjustments* that enable them to remain unhampered by emotional conflict and free from pathological symptomatology. This individual *confirms and follows a philosophy of living*; *finds satisfaction and fulfilment* in exercising and *expanding his potential*; and *establishes and maintains meaningful relationships* with others (Perko & Kreigh 1988).

Table 2 provides a list of central concepts, and essential and related criteria. The central concepts, essential criteria and related criteria are in italics.

The findings of this study were obtained from connecting isolated pieces of information by organising more than one interrelated concept to describe the structure and process of the model to assist psychiatric nurses in facilitating the mental health of adolescents abusing substances (Walker & Avant 2011). In the process, the unrecognised concepts were named and described (Gray, Grove & Sutherland 2017). The definition of the concepts ‘facilitation’ and ‘mental health’ were separately formulated, then finally, the central concept ‘facilitation of mental health’ was presented.

#### Definition of ‘facilitation’

In this article, ‘facilitation’ is defined as the dynamic interactive process between the psychiatric nurse and adolescents abusing substances, through which the psychiatric nurse guides adolescents abusing substances, in a way making it easier for them to cope with the challenges they encounter.

#### Definition of ‘mental health’

In this article, ‘mental health’ is defined as evidence that the adolescent has achieved a sense of maturity, exhibits positive emotions, has attained emotional intelligence, experiences subjective wellbeing and shows resilience.

#### Definition of ‘facilitation of mental health’

In this article, ‘facilitation of mental health’ is a dynamic interactive process through which the psychiatric nurse guides adolescents abusing substances, in a way making it easier for them to cope with challenging situations as evidenced by the adolescents’ achievement of maturity, the exhibition of positive emotions, attainment of emotional intelligence, the experience of subjective wellbeing, and showing resilience.

## Discussion

To obtain the desired outcomes of defining the central concept ‘facilitation of mental health’ of adolescents abusing substances, dictionaries and subject sources were used (Walker & Avant 2011). It was critical to define ‘facilitation of mental health’ since the concept is new in its context. The role-players, especially psychiatric nurses, should understand this concept comprehensively because they are at the front line in the facilitation of mental health of adolescents abusing substances assigned to their care when they get admitted to psychiatric institutions. The definition of ‘facilitation’ shares its components with the Theory for Health Promotion in Nursing (University of Johannesburg 2017) and other South African literature sources. Therefore, the conclusion can be drawn that the definition was derived and analysed in the South African context. Another crucial element in the description of the central concept ‘facilitation of mental health’ is that it was used to develop a model to assist psychiatric nurses in facilitating the mental health of adolescents abusing substances. The model case highlights the need for psychiatric nurses to assist adolescents abusing substances to make behavioural and emotional adjustments to achieve mental health.

## Constructing a model case

A model case is a concept that constitutes all the defining criteria of the concept (Walker & Avant 2011). The essential criteria of the concept ‘facilitation of mental health’ forms the basis of the model case (Rikhotso 2019).

Mr X is an 18-year-old boy who lives in a village in the Greater Giyani Municipality, situated around 20 km from the psychiatric institution where the study was conducted. He is the firstborn of two children and is living with his single mother and younger sister. His mother sells fruit to earn a living. He is still in Grade 10 because, in recent years, he has repeated every grade and sometimes does not even sit for examination because of hospitalisations on account of substance use induced psychosis. As a result of the scarcity of public transport in this area and the inaccessibility of police services to intervene, he is typically brought to the hospital in a hired vehicle, which is very expensive. He shared his story as follows:

The story, as explained to me by an old lady from the neighbourhood, is that Mr Smith (not the real name) impregnated my mother while she was still a teenager and lied to her that he was single. When she went to live with him in a nearby village, she discovered he was married and had children. For my mother, life became miserable, and she decided to return home. A few months later, when she was 4 months pregnant with me, she was proposed to by Mr Jones (not the real name), a man with whom she attended school and lived in the same village. Since he spent most of his time in Johannesburg, where he was working, he was not aware she was pregnant. My mother accepted the proposal and went back to Johannesburg with him. A month later, she returned home to Giyani, and she called him to say she was pregnant. Three months later, I was born. Mr Jones got the news that a baby boy was born, and he realised I was not his son. He rejected my mother, and she returned to her parents’ home with me. I had mixed feelings when I heard the story, but I was happy I finally got the truth.

At this stage, I was drinking beer, and every time I was drunk, I would confront my mother about her reasons for lying to me about my father. I got used to drinking, so I would drink more frequently, even alone, and bitterness started to build up in me. My academic performance declined; hence, I am still in Grade 10. My relationship with my mother became bad. My younger sister became fearful when I was around because I would hit her for no reason. At times, I would be irritable and aggressive. In my own corner, I was overwhelmed with feelings of hatred, anger and loneliness. My drinking habit resulted in me being admitted to this psychiatric institution. I am told I would become violent, beat my younger sister, swear at my mother, spend sleepless nights, and turn the radio to maximum volume. I’ve had numerous admissions to this institution. Upon discharge after every admission, I would vow I would not be admitted again, but when I get home, the same peers and circumstances push me to the edge, and I start drinking again. I could see that my future was bleak and wished to change my lifestyle, but it was not easy. I wished I could get some help, but did not know from who, where, and how.

By God’s grace, during my previous and last admission almost a year ago, I met Sister Margaret (not the real name), a psychiatric nurse in the ward in which I was admitted. To date, I am still in awe because for the first time in my admission history to this institution, I have spent many years at home without being readmitted. She introduced herself to me and offered to assist me. Although I was uncertain whether I would really get assisted, considering my recurrent admissions, I accepted her offer. At last, my question on where to go and from whom to get assistance was answered, though I still did not know how this would occur.

Together, we embarked on a journey of a dynamic interactive process that enabled me to open to her and share all my challenges regarding the use and abuse of substances, especially alcohol. This was possible after my conviction of her trustworthiness, and I became free to interact with and confide in her. I shared my addiction to drinking beer, my motives for continuing with the habit, the setbacks this has caused in my life, and the emotional, physical, and social challenges this has created with her. Although I did not have all the information, I was aware that my mind had been affected, and I therefore needed someone to guide and make it easier for me to become fully functional and for subjective wellbeing to unfold in my life.

She pointed out that from the information I gave her, I failed to fulfil my responsibilities as a young person and lacked a sense of maturity. Once I become willing to resume my responsibilities and become resilient, this dynamic interactive process would succeed. She further clarified that I am the one holding the key to my own future. Unless I take actions to enable me to improve my wellbeing and emotional intelligence, the process of our interaction would be of no use.

From that encounter to date, I cannot stop thinking about where I would be now if I did not have the opportunity to engage in the process of interacting with that sister. I am forever grateful for this because my life has not been the same since. I can relate socially to my mother and other people with whom I live. I manage to control my emotions and respond positively to challenges without being easily angered. Even my psychological state has improved; I am able to reason well and adjust to different situations I encounter. For a change, I have passed my examinations, and next year will attend Grade 11. I am very happy about this and never thought it would be possible. The changes that I already see in my life give me hope that I will end up living a life free from mental illness.

## Limitations of the study

The literature sources for the definition of the concept ‘facilitation’ were commonly obtained from the field of psychology.

## Recommendations

Recommendations are made for psychiatric nursing practice, education and research.

### Recommendations for psychiatric nursing practice

It is important to define ‘facilitation of mental health’ of adolescents abusing substances in the context of nursing, especially in the psychiatric wards where these adolescents are admitted and entrusted to the constant care of psychiatric nurses. Psychiatric nurses should have profound knowledge about the ‘facilitation of mental health’ of affected adolescents to assist them in making reasonable adjustments to their behaviour to achieve their goal of mental stability. The psychiatric nurses should attend workshops or receive in-service training on facilitating adolescents’ mental health to ensure they receive an effective and efficient service during their admission in the psychiatric ward and sustain their improvements after their discharge as this is not included in their formal training. The successful implementation of this concept could evoke a constructive debate in the field of nursing that would add to the body of knowledge in nursing practice within South Africa, as well as the global nursing community. This would improve the mental health of adolescents abusing substances based on the empowerment they would receive in handling the challenges they encounter. This will consequently improve mental health practice.

### Recommendations for nursing education

The definition of the central concept ‘facilitation of mental health’ of adolescents abusing substances could assist in teaching psychiatric student nurses – both at undergraduate and postgraduate levels as this is currently not in the curricula – about adolescent mental health and the benefits these adolescents could achieve from their mental health being facilitated. Having acquired this knowledge, student nurses would be empowered, thereby being able to render effective psychiatric nursing care to adolescents abusing substances.

### Recommendations for nursing research

In the process of analysing the central concept, the foundation was laid for the development of a model to facilitate the mental health of adolescents abusing substances. Psychiatric nurses’ evaluation of the model’s effectiveness progressively informed the need for further research on adolescents’ substance abuse and their mental health.

## Conclusion

The analysed concept acted as the foundation for developing a model for psychiatric nurses to facilitate the mental health of adolescents abusing substances. The nursing profession is enriched by defining the central concept since the ‘facilitation of mental health’ of adolescents abusing substances has never been defined before. It also illuminates psychiatric nurses’ added role in facilitating the mental health of adolescents abusing substances, which the South African Nursing Council has not acknowledged for inclusion in the scope of practice of psychiatric nurses.
